# Emotional distress and affective knowledge representation one year after the COVID-19 outbreak

**DOI:** 10.1371/journal.pone.0311009

**Published:** 2025-01-17

**Authors:** Laura Barca, Pierpaolo Iodice, Amine Chaigneau, GianLuca Lancia, Giovanni Pezzulo

**Affiliations:** 1 Institute of Cognitive Sciences and Technologies, National Research Council, Rome, Italy; 2 Movement Interactions Performance ‐ MIP, UR 4334, Le Mans Université, Le Mans, France; 3 CETAPS Lab., University of Rouen Normandy, Mont-Saint-Aignan, France; 4 Department of Psychology, Sapienza University of Rome, Rome, Italy; Polytechnic Institute of Coimbra: Instituto Politecnico de Coimbra, PORTUGAL

## Abstract

This study examines whether the detrimental effects of the COVID-19 pandemic on the affectivity of the population extend one year after the outbreak. In an online-mobile session, participants completed surveys (i.e., demographic characteristics, positive-negative affectivity, interoceptive awareness) and a similarity judgment task of triplets of emotional concepts, from which we derived 2D maps of their affective knowledge representation. Compared with pre-pandemic data derived from a comparable population, we report three main findings. First, we observed *enhanced negative affectivity* during the pandemic, but no changes in positive affectivity levels. Second, increased *self-reported interoceptive awareness* compared to pre-pandemic data, with greater attention to bodily sensations and adaptive aspects of interoceptive sensitivity. Furthermore, female participants reported higher scores than males on the questionnaire subscales of *Emotional Awareness* and *Attention Regulation*. Third, the effect of pandemic-related conditions is also apparent in the mental organization of emotional concepts, especially for female participants (i.e., reduced coherence in the organization of the concepts along the arousal dimension and more misclassification of concepts based on arousal) and participants who did not perform physical activity (a collapse of the arousal dimension). Some of the effects of the pandemic, thus, persist about a year after the outbreak. These results advise providing programs of psychological and emotional assistance throughout the pandemic beyond the outbreak, and that age-dependent gender differences should be accounted for to define tailored interventions. Physical activity might relieve pandemic-related stressors, so it should be promoted during particularly stressful periods for the population.

## 1. Introduction

The COVID-19 pandemic had a profound impact on our lives, both individually and collectively. One of the most significant effects of the pandemic has been on our emotional well-being. People around the world experienced a wide range of negative emotions, such as fear, anxiety, sadness, and anger. These emotions were caused by a variety of factors, including the threat of illness and death, the disruption of social and economic life, and the uncertainty about the future.

Contemporary constructivist views of emotions [1, 2] provide a useful framework to understand the impact of the COVID-19 pandemic on our affectivity and on our mental representation of emotional concepts. A cornerstone of constructivist views is that emotions are not innate or universal, but are instead constructed through social and cultural experiences [3]. This means that our understanding of emotions is shaped by our individual experiences, our beliefs and values, and the social and cultural norms that surround us. During the pandemic, our mental representation of emotions has likely changed, since certain emotions became more salient (e.g., fear, anxiety, and sadness)–as a consequence of the exposure to a constant stream of information focused on the negative aspects of the virus and its impact on our lives. Furthermore, the disruption of social norms (e.g., the norms around physical contact and social gatherings) has rendered emotional regulation more challenging, as we were no longer able to rely on the usual social cues and support systems that we used in typical conditions. In addition, the pandemic has created a great deal of uncertainty about the future, which has made it difficult to feel secure and optimistic, contributing to feelings of anxiety and fear–and to associated interoceptive processes. Given the constructive nature of emotional experience, how long does pandemic-related emotional distress last? Does it manifest at the level of the emotional domain in the broad sense, in the mental organization of the words we use to convey affectivity (i.e., emotional concepts)? The aim of this study is to evaluate emotional distress and affective knowledge representation of the Italian population, approximately one year after the initial outbreak. Italy was one of the first European countries to be severely impacted by the COVID-19 pandemic. The nation implemented stringent containment measures early on, leading to a prolonged period of lockdown and significant socioeconomic disruption. These factors created a unique context for studying the psychological effects of the pandemic and its long-term consequences.

### 1.1 The impact of the COVID-19 pandemic on emotional well-being

Starting from March 2020, Italy has been strongly hit by the COVID-19 pandemic and has seen a progressive increase in restrictive measures to contain the spread of the virus. Several studies reported a detrimental effect of the pandemic on the affectivity of the population, typically with a reduction in experiencing positive affectivity and an increase in negative affectivity. One month after the outbreak, a large portion of the Italian population was perceiving a form of psychological distress [4]. During the lockdown, depressive, anxiety, and stress-related symptoms increased in the general population (particularly in females) [5], as well as sleep difficulties [6]. Studies conducted in other countries report comparable findings. A cross-sectional study conducted in Spain reported that after 8 weeks of lockdown ‐ with total home confinement ‐ the population experienced an increase in negative affectivity as feeling ‘upset’, ‘scared’, and ‘distressed’, together with a reduction in positive affect as ‘exited’, ‘active’, ‘interest’ [7]. The increase in negative affectivity was higher for females and the younger groups of participants (i.e., < 36 years, among 36–60 years of age) compared to the older group (i.e., > 60 years). Likewise, in China higher emotional distress has been reported by the younger population and by females, the latter experiencing an increase in anxiety, stress, and depression [8]. Studies in the UK reported an increase in the diagnosis of eating disorders and self-harm episodes among teenage girls in the 2 years since the onset of the COVID-19 pandemic [9, 10] (see also Barca, Maisto and Pezzulo, 2023, for a theoretical-computational account of food restriction and non-suicidal self-harm behaviours in adolescence) [11].

One of the factors affecting emotional distress during a pandemic might be the type of containment measures adopted to limit the spread of the disease [12]. Cross-country comparisons indicate that as the severity of the containment measures increases, the level of emotional discomfort might also increase. Eichenberg et al., (2021) [13] compared the emotional well-being of the Italian, German, and Austrian populations. The survey study was conducted during the first lockdown when the containment measures largely vary between those countries. Since, among European countries, Italy was immediately hit very hard with a high number of victims and a high spread of the infection, it soon adopted very restrictive measures (e.g. the suspension of didactic activities of all types and levels; the closure of non-essential activities (except for grocery stores, pharmacies, and shops for necessities and essential services), the prohibition of any form of gathering of people in public places or places open to the public, and the prescription to maintain an interpersonal distance of at least one meter and wearing masks) and for a longer period to these other countries where, for example, people were allowed to leave their home for work-related reasons. In Eichenberg et al., (2021) [13] study, the Italian sample reported higher levels of anxiety and negative affect since, as the authors suggested, they have been exposed to higher levels of physical stressors (e.g., the danger of life due to a greater spread of the virus), social stressor (e.g., social distancing, suspension of gatherings as weddings and funerals) and also economic stressor (e.g., fear of losing the job, reduction of the income).

Although several studies address the emotional impact of the early stage of the pandemic (see for example Santomauro et al., 2020) [14], some aspects deserve further attention. A topic that remains unaddressed, for example, is how long the emotional discomfort persists and varies according to the different stages of the pandemic. The 2003 SARS outbreak demonstrated that the emotional burden of epidemic events might leave a trace on the mental health of the population years after the outbreak, with the quarantining being predictive of high levels of depressive symptoms [15]. The COVID-19 pandemic did not have a linear trend, with a definitive resolution after the first emergency and the adoption of severe measures to contain the spread of the virus. Rather, the pandemic had a cyclical trend with acute phases alternating with phases of reduction of the virus’ bitterness and the number of victims, coupled with times of easing and subsequent tightening of containment measures. Thus, even though the emergency phase experienced in March 2020 might be over, the population has been subjected to physical, social, and economic stressors for a long period, together with an additional stressor due to the uncertainty of the epidemic, which might foster the state of discomfort beyond the outbreak. Hence, it is crucial to assess whether the emotional distress observed during the COVID-19 outbreak [4, 16] persists beyond the emergency, to implement appropriate support and intervention policies.

To address this issue, we conducted an online study to explore emotional well-being during the period from May to June 2021 (beyond the Italian emergency phase). At the time this study was conducted, Italy reported a total of 121,177 confirmed deaths due to the COVID-19 virus (source https://ourworldindata.org/, (The data was taken from the ’Our World in Data’ website, a project of the Global Change Data Lab, a non-profit organization based in the United Kingdom (Registered Charity Number 1186433).The data was gathered from four sources: Specialized institutes; Research articles; International institutions or statistical agencies; Official data from government sources–for example, the COVID-19 vaccination and testing datasets compile the most recent official numbers published by governments and health ministries worldwide)). Some of the restrictions were lifted (e.g., schools reopened). However, others were still in effect (e.g., wearing masks indoors, and social distancing not mandatory but recommended), and overall the populations have been subjected to the pandemic-related stressors for about 14 months.

### 1.2 The current study

We administered a self-report questionnaire to evaluate participants’ *affectivity*, a self-report questionnaire to evaluate *interoceptive awareness*, and an *emotional concepts similarity judgment task* which we recently developed and described in Barca, Candidi, Lancia, Maglianella, and Pezzulo (2022) [17]. The study described by Barca et al., (2022) [17], conducted prior to the pandemic in January 2020, serves as a valuable baseline for our investigation into the impact of COVID-19 on emotional and interoceptive dimensions within the Italian population. By employing the same surveys and emotion similarity task, we can effectively assess the potential changes induced by the pandemic.

The first question addressed in the study is whether the Italian population exhibited emotional distress in a period beyond the pandemic emergency. To address this issue, we collected online self-report measures of the population *affectivity* through the Positive and Negative Affectivity Scales questionnaire, which measures the tendency to positively or negatively evaluate life events [18]. Overall, we expected a reduction in positive affectivity and an increase in negative affectivity compared to the pre-pandemic period [17].

We also considered participants’ *interoceptive awareness* (i.e., a self-reported measure of the attention toward one’s bodily signals) as measured by the Multidimensional Assessment of Interoceptive Awareness questionnaire [19]. Interoception refers to the sensing of the physiological conditions of the body [20], which is key to homeostatic and allostatic regulation [21, 22], consciousness [23], well-being, emotional and affective states [24], and the processing of emotional stimuli [25, 26]. An accurate perception of bodily signals facilitates emotion regulation via cognitive reappraisal (i.e., a re-interpretation of the meaning of a negative situation so that it no longer feels negative), with a down-regulation of affect [27]. Rather, impaired interoceptive processing could be linked to various psychopathological conditions [28–32].

Considering that from the early stages of the pandemic the population has been asked to monitor their bodily conditions (e.g., body temperature and respiratory difficulties, such as cough and painful throat) for the identification of COVID-19 symptoms, such greater attention to bodily conditions might have fostered an increase in interoceptive awareness. However, greater attention to bodily signals might not necessarily be beneficial. Beneficial aspects of interoceptive sensibility, as a *mindful attention style* towards interoceptive cues, might be distinguished by an *anxiety-driven attention style*, which has been associated with somatization and anxiety disorder [33]. In keeping, a high score on the Noticing scale of the MAIA questionnaire (comprising items that specifically measure the attention towards the signals from the body) has been associated with maladaptive aspects of interoceptive awareness [34], and recently with an increase in symptoms of anxiety and depression under the ‘mild lockdown’ adopted in Japan [35]. Here, we wanted to test which aspects of interoceptive awareness have been primarily affected by the pandemic, expecting the Italian population to report higher scores on maladaptive aspects of interoceptive awareness to the pre-pandemic period [17].

A second question addressed in this study is whether pandemic-related emotional distress also manifests at the level of affective knowledge representation. Some studies highlighted the malleability of conceptual knowledge to different contextual conditions, as the first lockdown of the pandemic [36–38]. The emotional features of words–such as the ratings of affective valence and physiological arousal ‐ are not stable properties of the corresponding concepts. Rather, they are sensitive to contextual conditions, such as the linguistic context (as manifest in list composition effects during experimental studies) and the social conditions in which they are tested. When rated during the pandemic, pandemic-related words such as ‘cough’ were rated with heightened arousal than words not related to the pandemic as ‘cumin’, these latter having similar ratings to the pre-pandemic dataset [38]. The valence ratings were less permeable to contextual variations, but the authors reported an ‘increased positivity’ of words such as ’kiss’ or ‘hugs’, under a possible ’nostalgia boosting effect’ due to social distancing. The flexibility of conceptual knowledge is one of its core features, and the organization of concepts varies with the inclusion of new entries as the COVID-19 one [36, 39]. In a free listing study addressing the characteristics of disease concepts during the first lockdown, the ‘COVID-19’ concept was more emotionally connotated than other disease concepts as ‘tumor’ or ‘fluid’, by evoking more frequently negative-emotions terms such as ’fear’ [36].

Here, we tested whether the pandemic-related contextual conditions have an impact on affective knowledge representations, with a specific focus on the mental organization of emotional concepts, along the two dimensions of affective valence and physiological arousal. Different from the aforementioned survey studies [36, 38] we gathered an *implicit measure of participants’ conceptual knowledge* as measured by a similarity judgment task, conducted online via mobile phones. Participants were asked to pair triplets of emotional concepts (varied according to affective valence and physiological arousal) according to their similarity, by tapping on the response option on the screen. A similar procedure has been previously described by Barca et al., (2022) [17], where behavioral and kinematic measures collected with PCs were used to compute a similarity index between concepts–and then derive 2D topographical maps of emotional conceptual knowledge. The maps allowed the characterization of the ’emotional space’ of the participants. Emotional concepts were arranged in a 2D topographical map, which represents them along a continuous spectrum encompassing the two dimensions of valence and arousal [40–42]. The fact that emotional concepts span across a large portion of the map suggests that people are attuned to subtle variations within these two dimensions, rather than perceiving emotions as strictly positive or negative, or highly aroused or calm. Individuals with reduced positive affectivity exhibited a narrower distribution of the concepts, especially regarding positive emotions (e.g., ‘social’) and emotions with reduced arousal (e.g., ‘respectful’). The close proximity of certain emotions on the map suggests potential for easier transitions between them (see also Cowen & Keltner, 2021) [43] and for the possibility of experiencing seemingly opposite emotions simultaneously (or ‘mixed emotions’). Experiencing mixed emotions, like feeling happy and sad at once, is not only common [44] but also a sign of greater emotional complexity and adaptability [45]. Relevant to the current study, a recent investigation found a correlation between experiencing mixed emotions and increased adherence to virus prevention measures, such as social distancing, during the COVID-19 pandemic [46]. We expect the pandemic-related conditions to shape the mental representation of emotional concepts, with greater sensitivity of the arousal dimension, which we hypothesize to be more malleable to changes in contextual and physiological conditions.

## 2. Materials and methods

*Ethical approval*. The experimental procedure has been approved by the Ethical Committee of the ‘Istituto Nazionale per le Malattie Infettive Lazzaro Spallanzani IRCCS’ (Parere 88_2020).

### 2.1 Participants

A snowball convenience sampling strategy was used to recruit participants through social media (i.e., Facebook). Inclusion criteria were: (i) working in Italy and (ii) being fluent in Italian. The exclusion criteria were: have Covid-19 at the time of testing; have been affected by Covid-19 in the last three months. Participants reported to be right-handed and with normal or corrected to normal vision. The disproportion in participants’ gender might reflect a gender bias in the distribution of students among whom the link for the experimental session has been disseminated, or in the willingness to participate in the online research. A total of 89 participants took part in the online task.

We calculated the power of the behavioral study Barca et al., (2022) [17] and employed that value as a reference point for our current study. The decision is guided by both theoretical and methodological considerations. The study by Barca et al. (2022) [17] provides a comprehensive assessment of emotional and interoceptive dimensions in the Italian population prior to the pandemic. Their findings establish a baseline understanding of these dimensions, which is essential for evaluating potential changes induced by the pandemic. Additionally, employing the same surveys and emotion similarity task in both the pre-pandemic and pandemic studies allows for a consistent and comparable assessment of emotional and interoceptive dimensions. By using the pre-pandemic data as a reference point, we aim to gain a deeper understanding of how the COVID-19 pandemic may have impacted emotional and interoceptive experiences in the Italian population.

A post hoc power calculation test performed on the pre-pandemic sample, indicated that a sample of 30 participants was adequate to have a statistical power of 0.63 (p < 0.05). Based on this evidence, we believe the current pandemic sample of 89 participants is adequate to provide useful evidence. Below, we provide a description of the pre-pandemic study group.

#### 2.1.1 Characteristics of the pre-pandemic sample (Barca et al., 2022)

The study by Barca et al. (2022) [17] involved a sample of 30 healthy adults, gender-balanced (15 female), aged 21–37 years old (mean age 24.5 ± 2.7 years). The participants were university students recruited at the Faculty of Psychology of the University of Rome ‘La Sapienza’ through in-person advertisements. They were right-handed and with normal or corrected to normal vision.

### 2.2 Mobile phone experimental session

The research described was an online experiment conducted on the web. Participants engaged in the task using their mobile devices. The web interface was developed using JavaScript, mainly based on the *jsPsych* (v.6.3.0) library [47, 48] (The entire experiment, including the task, code, and libraries used, can be accessed at https://github.com/AmineChaigneau/js_psy_task). This library allows for various behavioral experiments, including tasks that measure response time in stimulus-response scenarios. However, it should be noted that response times measured through web processes might not be entirely precise and typically exhibit a consistent variance of approximately 10–40 [49].

**Fig 1:**
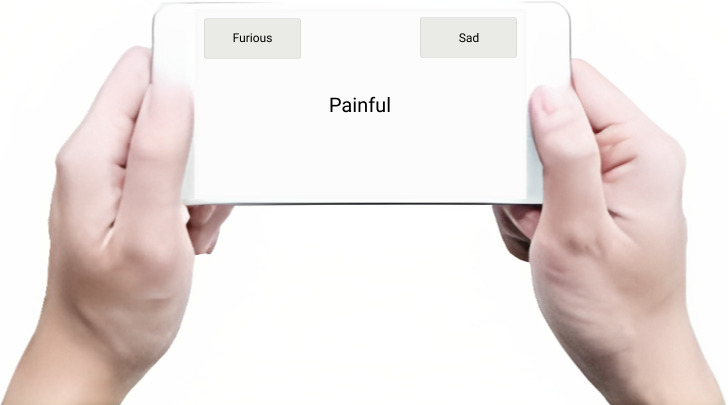
Illustration of how participants were asked to hold the mobile phone during the execution of the emotional concepts similarity judgment task.

The experiment consisted of a single session lasting between 25 and 30 minutes. Participants were instructed to complete the task only once. The experiment was conducted in full-screen mode (*JS Fullscreen API*). Participants were given the option to choose between French and Italian as the language for the experiment (data from the French sample is not discussed in this context). Participants were asked to hold their phone horizontally and use their two thumbs to select answers, positioned on the left and right of the screen respectively (see Fig 1).

At the beginning of the session, participants were provided with the information necessary to provide Informed Consent to participate in the experiment and Informed Consent to data processing. After having consented to the participation and data processing (by clicking on the appropriate box), participants were asked to provide demographic information and details about their living conditions. Specifically, they were queried about their employment status (such as permanent employment, fixed-term position, freelance work, trading, university student, or unemployed), household composition (e.g., living alone, with a partner and at least one child; without a partner but with at least one child; the presence of a minor child), housing characteristics (e.g., whether they had a garden or not), geographical location, and their engagement in sports or physical activities (e.g., running, soccer, yoga). These questions were useful to investigate the effect of specific stressors (e.g., unstable working conditions) or life conditions that might mitigate containment measures (e.g., having a garden or performing physical activity). After filling out the form, participants were provided with a training session to familiarize themselves with the similarity judgments task, which will be fully described below. Finally, they completed the PANAS [18] and the MAIA [19] self-report questionnaires.

#### 2.2.1 Similarity judgment task

*Materials*. We used the materials described in [17], a list of 24 emotional words varied according to the Valence and the Arousal ratings from the Montefinese database [50].

*Procedure*. Participants had to pair emotional concepts based on their similarity, by tapping on the mobile screen. Triplets of emotional words that varied for Arousal (high/low–‘terrified/respectful’) and Valence (pleasant/unpleasant–‘brave/angry’) were presented in each trial. The ‘target’ concept (e.g., ‘lonely’) was centrally presented, and the two response options (e.g., ‘terrified’ and ‘brave’) were displayed in the top corners of the screen. Participants were asked to respond as quickly as possible by tapping on one of the two response buttons located at the top left and right of the screen (the size of the buttons was defined as the relative width and height of the screen size). Once the task was initiated, participants performed 288 randomized trials (j*sPsych*.*randomization* function), making 288 pairing decisions. Each trial consisted of a triplet of stimuli. The 24 stimuli have been fully rotated in the tree position (central, top left, and top right) according to the valence dimension, giving rise to 6 conditions of 48 trials each. In the case of the presentation of a target with positive valence, for example, we considered three semantic contexts: *full congruence* (if both response options conveyed a positive valence), *partial congruence* (if one response option carried a positive valence, while the other conveyed a negative valence ‐ presented either on the left or right sides, balanced), and *no congruence* (both response options conveyed a negative valence).

The data collection was preceded by a practice section of 6 additional trials. To begin each trial, participants tapped on the /START/ button located at the bottom-center of the mobile screen. Then the three stimuli appeared and remained on the screen until the participant’s response. Response Choice and Response Times (i.e., from when participants pressed /START/ until they tapped the response option) have been recorded and used to compute the similarity index between concepts, and the 2D maps. At the end of the experiment, all the data were sent to a secure database developed under the python using Django library (v 3.1).

#### 2.2.2 Self-report questionnaires

The *Positive and Negative Affectivity Schedule* (PANAS) [18] has been used to measure the tendency to positively or negatively evaluate life events. It comprises two scales measuring positive and negative affect, which may be described as general mood dimensions. Participants rate their current affectivity (i.e., state affect) using a five-point rating scale, ranging from 1 (‘very slightly or not at all’) to 5 (‘extremely’) on each of 20 words that describe different states, emotions, and feelings (e.g., ‘interested’, ‘distressed’, ‘nervous’, ‘proud’). We used the Italian version of PANAS, whose validation has been described in [51] and confirms that it is a reliable and valid measure of self-reported affect.

The MAIA [19] has been used to measure *interoceptive sensibility*. The questionnaire comprises 32 items, which measure eight different facets of interoceptive sensibility: *Noticing* (being aware of body sensations), *Not-Distracting* (being inclined to not distract or ignore painful or uncomfortable sensations), *Not-Worrying* (inclination to not be emotionally distressed by uncomfortable sensations), *Attention Regulation* (paying attention to and controlling attention on body sensations), *Emotional Awareness* (being aware of the connection between emotions and body sensations), *Self-Regulation* (regulating distress through paying attention to body sensations), *Body Listening* (purposefully listening for insight from the body) and *Trusting* (experiencing trust with and safety in the body). Higher scores indicate greater sensitivity to signals from the body.

We used the Italian version of MAIA, whose validation for the Italian language has been described in [52].

### 2.3 Data analysis

*Bayesian estimation of parameters* [53–55] has been used to test for differences in the PANAS and MAIA scores between the pandemic period and pre-pandemic. The Bayesian estimation approach produces information about the magnitude of the parameters of the posterior distribution (e.g., means, standard deviations, effect sizes) (The difference of the means, relative to the average standard deviation of the groups.), and their difference, and can quantify support for both the alternative and the null hypothesis. The Bayesian inference has been computed in the programming language R (R Development Core Team, 2011, version 4.0.2) and the Markov chain Monte Carlo (MCMC) sampling language JAGS with the BEST package [56]. The MCMC process generates a random sample of credible parameter values of the posterior distribution (we used a default sample size of 100,000). We used the 95% Highest Density Interval (HDI) as a measure of the credibility of the parameters, where every value inside the interval has higher believability than any value outside the interval. We established a Region of Practical Equivalence (ROPE) (The ROPE indicates a small range of value that are considered to be equivalent to the null value (Kruschke, 2011)) on the distribution of effect sizes, ranging from -0.1 to 0.1. According to Cohen (1988) [57], an effect of size of 0.1 is small, thus if the majority of the credible values fall within the ROPE, the null value is credible and accepted for practical purposes [54].

*Bayesian correlation analysis* [58] has been used to explore the pattern of inter-correlation between the scores of the eight subscales of the MAIA questionnaire. The Bayesian Correlation Pairs have been computed in the programming language R (R Development Core Team, 2011, version 4.5.1) with the ‘correlation’ package [59, 60]. The analysis produces a Bayes Factor (BF), a measure that indicates the likelihood of the data under the null hypothesis and under the alternative hypothesis. BF values greater than 1 are considered evidence in favour of the alternative hypothesis, and values smaller than 1 are considered evidence in favour of the null hypothesis.

## 3. Results

### 3.1 Demographic characteristics

A total of 89 participants took part in the study (54 female, 35 male), with an average age of 35 years (age range 18–69) (no exclusions were made). The data acquisition was conducted online, in complete autonomy by the participants, and not everyone completed all the tests of the session (when we deal with the individual tests we will specify how many participants completed them).

The distribution of the *working conditions* indicates that the majority of them had a job (29% permanent employment, 27% freelance). Others (12%) were university students or were unemployed (13%). Few participants had fixed-term contracts (3.4%), some were traders (1.2%), and 13.5% of participants did not provide such information.

As for the *household condition*, a total of 17 respondents declared living alone (19%), and 26 were cohabiting with their partner and at least one child (29%). Another group of 20 respondents declared living alone with at least one child (22.5%). Most of the participants, therefore, declared to have at least one minor child and this might be considered an additional stressor, particularly in the period in which the schools have been closed and for those in smart working. The last group of 26 participants stated that they had a different situation from the options provided (29.2%).

Considering the *housing* characteristics, the majority of our sample have a garden (82%), which potentially allowed them to spend some time outside their home during the confinement. This is an important feature of the current sample, since having an outdoor environment may have partially alleviated the burden of the home-confinement and other restrictions.

The vast majority of the participants performed *physical activity* (72%), either running (47.2%) or soccer (24.2%).

Concerning the *geographical location*, most of the participants were located in the southern (49%) and central regions (26%) of Italy. One-fourth of the current sample (25%) was located in northern Italy, the region that was hit the hardest by the pandemic.

Eight participants declared to be positive for COVID-19 when they performed the task.

### 3.2 Positive and negative affectivity (PANAS)

**Fig 2:**
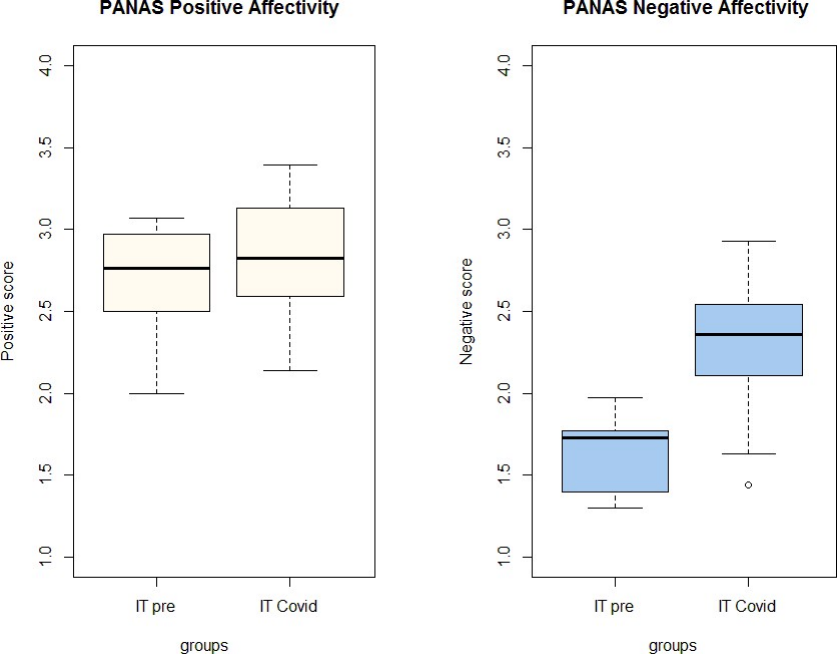
Boxplot depicting the PANAS scores. IT pre: pre-pandemic dataset by Barca et al., (2022) [17]; IT Covid: pandemic dataset.

A total of 70 participants filled in the questionnaire, and we performed a by-items analysis based on the available data (i.e., we first computed the mean score of the available sample on the twenty items of the PANAS scale, then we subjected the values to Bayesian parameters estimation). The PANAS data collected in Barca et al.’s study (2022) [17] has been used as a comparison with the current dataset as a proxy for the affectivity values of the pre-pandemic period. Fig 2 reports the average values of positive and negative affectivity scores of the pandemic (based on 70 participants) and pre-pandemic samples (based on 30 participants).

Inspection of Fig 2 suggests an overall between-groups similarity in positive affectivity before and during the pandemic (left panel). Differently, the scores for negative affectivity (right panel) appear to be higher in the pandemic sample.

In the pre-pandemic sample, both the scores of positive and negative affectivity were in the normal range (27.1 and 16.4, respectively), and close to the normative data reported by [18], and the Italian validation of Terracciano et al. (2003) [51]. The pandemic sample scores higher on negative affectivity (22.9 ± 0.4), which is greater than 1 standard deviation of the normative sample. The negative affectivity scores are higher on all items on the scale, except one (’guilty’). Particularly high are the scores of the items ‘nervous’, ‘irritable’, and ‘frightened’, which are among those most correlated with measures of anxiety [18]. The score of positive affectivity (25.6 ± 0.4) is lower than the normative sample, but within normal range.

Bayesian parameter estimation [53, 56] has been used to test for possible differences and their credibility. The results of the analysis confirm that the positive affectivity scores of the pandemic and pre-pandemic samples are not credibly different (see the S1 Appendix for a full description of parameter estimation). Parameter estimation of the scores of negative affectivity indicates that the mean of the pandemic sample is credibly larger than the mean of the pre-pandemic sample, and the standard deviation of the pandemic sample is also credibly larger than the standard deviation of the pre-pandemic sample.

Considering gender differences, negative affectivity scores are placed at the upper edge of the normative range (Watson et al., 1988a) for both males (23.16 ± 0.5) and females (22.84 ± 0.5), and higher than the score of the pre-pandemic sample (males 15.3 ± 0.2; females 17.7 ± 0.3). The scores on positive affectivity (males 28.4 ± 0.4; females 28.1 ± 0.4) are close to the pre-pandemic sample (males 28.2 ± 0.4; females 26 ± 0.3).

Parameter estimation has been performed separately on male and female participants, to test for variation in affectivity according to gender. In the context of positive affectivity scores, no credible differences emerge in the mean and standard deviation of females’ pandemic and pre-pandemic samples, as for the males’ pandemic and pre-pandemic samples. Differently, in the context of the negative affectivity scores, both the mean and standard deviation of the males’ pandemic sample are credibly higher than the values of the males’ pre-pandemic sample. The sample means of the female groups are also different, but the posterior distribution reveals great uncertainty in the estimate of the difference of means.

To summarize, during the pandemic the score of negative affectivity increased on average; however, the variability across subjects increased as well, indicating that for some people the self-report negative affectivity increased and for others decreased. More specifically, negative affectivity increased on average in males (as the within-group variability) but the estimate for females was very uncertain, preventing drawing conclusions. As regards positive affectivity, we cannot draw any conclusions because the estimates of the parameters are very uncertain.

### 3.3 Interoceptive sensibility (MAIA)

A total of 87 participants filled in the questionnaire. The MAIA scores of the current pandemic sample and the pre-pandemic dataset (based on 30 participants) are shown in Table 1. Note that in (Barca et al., 2022) [17] we used the MAIA version 2 [61], in which five items were added to some scales. To compare the two datasets (pre-pandemic and pandemic) we eliminated the five items mentioned above from the data gathered in (Barca et al., 2022) [17] dataset.

**Table 1:** Mean and interquartile range (in bracket) on the MAIA.

	Pre-pandemic period			Pandemic period		
MAIA subscale	ALL	Female	Male	ALL	Female	Male
**Attention Regulation**	2.97 (.93)	2.86 (.93)	3.08 (.79)	3.09 (1.28)	3.23 (1.43)	2.83 (1)
**Body Listening**	3.14 (1)	3.13 (1)	3.16 (1)	2.92 (1.34)	2.99 (1.34)	2.80 (1.67)
**Emotional Awareness**	3.53 (.95)	3.49 (1)	3.56 (.8)	3.60 (1.15)	3.75 (.8)	3.33 (1.2)
**Not-Distracting**	3.14 (1)	3.33 (.83)	2.96 (.66)	2.85 (.33)	2.90 (.66)	2.76 (.33)
**Noticing**	3.38 (1.1)	3.40 (1.25)	3.37 (.75)	3.43 (1)	3.51 (1)	3.30 (1.25)
**Not-Worrying**	2.54 (1)	2.38 (1.33)	2.71 (.66)	3.30 (1.33)	3.36 (1.33)	3.19 (1.67)
**Self-Regulation**	2.95 (1.2)	2.77 (1.13)	3.12 (1.25)	3.06 (1.5)	3.13 (1.25)	2.93 (1.25)
**Trusting**	3.47 (.92)	3.18 (1)	3.76 (.67)	3.69 (1)	3.75 (1)	3.59 (1)

The questionnaire scores have been subjected to Bayesian parameter estimation [56]. The difference between the means and standard deviation of the MAIA score of the pandemic and the pre-pandemic sample is small, and the posterior distribution indicates great uncertainty in the estimate.

Inspection of Table 1 suggests that such uncertainty in the estimates might be due partly to between and within-group variability. In the pandemic sample, for example, female participants have higher scores than males on the *Emotional Awareness* (score difference of 0.42) and *Attention Regulation* subscales (score difference of 0.39). Such a difference is reduced in the pre-pandemic sample, and in other subscales of the questionnaire, the score difference is in the opposite direction. So, for example, females reported lower scores than males in the *Trusting* subscale (score difference of -0.58).

Parameter estimation of female and male MAIA scores during the pandemic indicates that the mean of females’ scores is credibly larger than the mean of the male participants. The estimate of variability has high uncertainty. In the pre-pandemic sample, although the mean scores are different ‐ the mean of females is smaller than the mean of males ‐ the posterior distribution indicates great uncertainty in the estimates.

Parameter estimation of the MAIA score of male participants of the pandemic and pre-pandemic sample indicates that the mean score during the pandemic is different (here smaller), but the posterior distribution reveals great uncertainty in the estimate (as for the variability). In the context of female data, the mean score of the pandemic sample is credibly larger than the mean score of the pre-pandemic sample. As for the variability, the posterior distribution reveals great uncertainty in the estimate.

Overall, these results indicate that during the pandemic, females’ MAIA scores are on average higher than males’ MAIA scores. Moreover, females’ MAIA scores during the pandemic are on average higher than females’ MAIA scores in the pre-pandemic sample. For other comparisons, there was not enough precision in the estimates to accept or reject the null value.

Next, we explored the pattern of inter-correlation between the scores of the eight scales of the MAIA, performed with Bayesian correlation analysis [58].

In the reanalysis of the pre-pandemic dataset [17], 7 out of the 28 possible paired correlations were significant with BF > 10, indicating strong evidence in favor of the correlation. The percentage of internal correlation in the pre-pandemic dataset (25%) is smaller than in other studies (e.g., Calì et al., 2015 reported a correlation rate of 57%) [52], possibly due to methodological differences (i.e., sample differences, Bayesian correlation vs Pearson correlation); however, the overall correlation pattern resulted comparable. The most related subscales were *Attention Regulation* and *Trusting* (BF > 1000), *Emotional Awareness* and *Body Listening* (BF > 1000), and *Noticing* with *Emotional Awareness* (BF = 216.21), *Self-Regulation* and *Body Listening* (BF = 124.93) with Bayes Factor indicating extreme evidence for H_1_ (i.e., a strong correlation between the subscales).

In the pandemic dataset, the pattern of correlations substantially increased, both in terms of the inter-correlation between the scales (15 out of 28 paired correlations were significant, that is 53.6%) and the strength of the correlations. Specifically, the *Noticing* subscale was extremely correlated with *Emotional Awareness*, *Body Listening* (both BF > 1000), *Attention Regulation* (BF = 366.15), and *Self-Regulation* (BF = 29.3). The *Noticing* subscale was suggested to reflect a maladaptive aspect of interoceptive sensibility [19] and it has been recently associated with an increase in symptoms of anxiety and depression during mild lockdown [35]. However, here, the rho score suggests a *positive relationship* between the awareness of body sensations and adaptive aspects of interoceptive sensibility, as the capacity to regulate attention to numerous sensory stimuli (*Attention Regulation*) and the awareness of mind-body integration (*Emotional Awareness*, *Self-Regulation*, *Body Listening*).

*Not-Distracting* and *Not-Worrying*, which compose the ‘Emotional Reaction and Attentional Response to Sensations’ dimension were not associated with other subscales [52], and were negatively related between each other (rho = -.39, BF = 130.46), indicating that distracting from unpleasant body sensation might be related with a reduction in emotional distress.

An increase in *Attention Regulation* was associated with an increase in *Trusting*, *Self-Regulation*, *Body Listening* (all BFs > 1000), and *Emotional Awareness* (BF = 257.84). Thus, an increase in the ability to sustain and control attention to body sensations was also accompanied by an increase in active listening to the body for insight and confidence in those signals, in the ability to regulate psychological distress by attending to body sensations, and in the awareness of the connection between body sensations and emotional states. *Emotional Awareness* was related to *Body-Listening* (BF > 1000), *Self-Regulation* (BF > 1000), and *Trusting* (BF = 34.13). Finally, *Self-Regulation* was related to *Body Listening* and *Trusting* (BFs > 1000), and *Body Listening* was related to *Trusting* (BF = 104.07).

In sum, we found that during the pandemic period, there was an increase in (self-reported) interoceptive awareness, with greater attention to bodily sensations and an increase in adaptive aspects of interoceptive sensitivity.

### 3.4 Topographical maps of emotional concepts

A total of 89 participants completed the similarity judgment task. Out of the original 89, 13 participants were discarded for showing an average response time (RT) lower than 1.5s. Subsequently, we filtered out the trials with too fast or too slow, setting the thresholds respectively to lower than 1 second (461 out of 20880 trials, or 2.2% of the trials) and higher than 10 seconds (940 out of 20880 trials, or 4.5%). We ended up with a total of 76 participants and 19469 trials. We visualized the mental representation of emotional concepts, based on the behavioral indices of the similarity judgment task performed during the pandemic.

Technical issues occurred during the online administration of the task causing an uneven presentation of the trials. Specifically: one trial (out of 288 trials) was repeated four times, while two other trials were skipped; we took the averaged index of the trial repeated multiple times, while the missing indices (2 out of 276 possible pairs, ~0.7% of the total indices) where substituted with the mean index for that particular participant. The limited number of these missing data does not affect our subsequent analysis.

We computed and plotted a *similarity index* (i.e., a measure of the similarity) between emotional words on a 2D planar space, which represents the words as nodes and their distance in space as (inverse) similarity. For each trial, we computed the ‘similarity index’ between two pairs of words: the target T and response A, and the target T and response B. The similarity index of TA and TB is calculated by combining information about the selected Response Choice and Response Time. When A was the selected response, we calculated the similarity index of TA as (S) = 0.6 + 0.4 * (RT), where RT is normalized to the [0, 1] range and the value 0.6 is an arbitrary weight given to the correct Response Choice. This implies that the similarity TA is greater if A is the selected response (Response Choice), and if the response is selected with faster reaction time. Finally, we set TA + TB = 1 for each trial, hence we could calculate TB = 1 ‐ TA. Note that the similarity index used here is slightly different from the one used in (Barca et al., 2022) [17]. This is because in the pandemic dataset the measure of Area Under the Curve (AUC) used in Barca et al., (2022) [17] was not available (due to the mobile data acquisition adopted here). To make the two datasets comparable, we removed AUC information from the pre-pandemic calculation.

**Fig 3:**
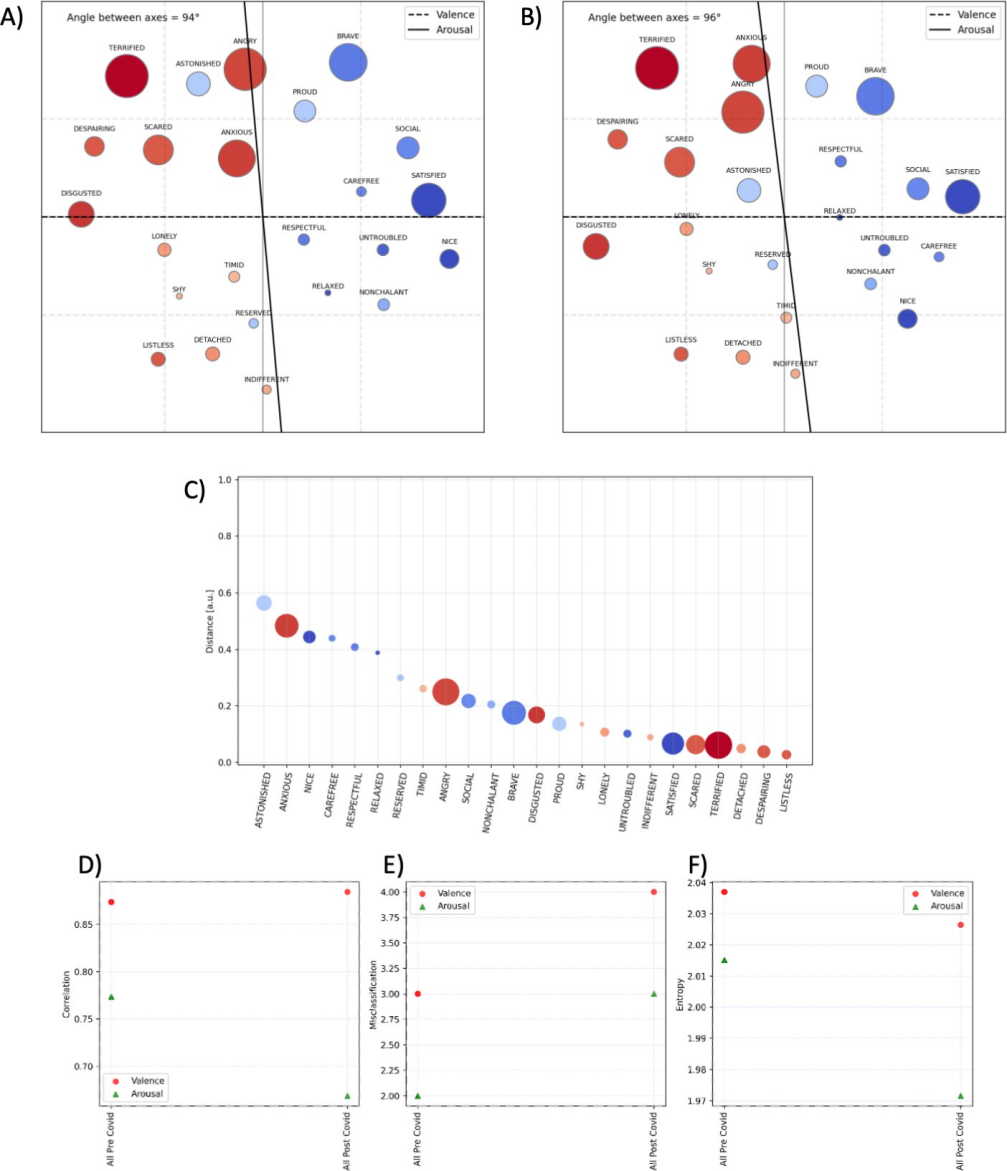
Two-dimensional map of emotional concepts of the pre-pandemic (panel A) and pandemic period (panel B). Color coding indicates Valence (blue: pleasant; red: unpleasant) and the size of the node indicates Arousal associated with the emotion (big: high arousal; small: low arousal). Each axis is the line centered in the origin (set at the barycentre of the nodes/stimuli) with an orientation that maximizes the spread of the projection of the arousal (or valence) values of the stimuli on the axis (i.e., the Pearson correlation between the axis and the arousal (or valence) values of the stimuli). Panel C: Distance between the location of each emotional concept in the map of the pre-pandemic and pandemic sample (i.e., the Euclidean distance of each item in the valence-arousal plane of the two groups). Panel D: Pearson correlations between the scores of valence or arousal and the projections on the two respective axes. This and the following indexes are calculated for the maps of different participant groups, pre-pandemic and pandemic. A higher correlation indicates that emotional concepts are better aligned along the axis. Panel E: Misclassification of emotional concepts for the different participant groups. Panel F: Entropy of the concepts across the valence and arousal dimensions. Entropy is calculated as the spread of concepts, separately for each axis, and is an index representing whether nodes span a large (high entropy) or small (low entropy) portion of the dimension.

As participants encountered the same pair of stimuli (e.g., TA or TB) multiple times and in different positions during the experiments, the final similarity index was the mean of the similarity scores calculated for each trial; missing pairs (due to missing data or filtered out trials) are substituted with the mean similarity index for that plot. The Kamada-Kawai algorithm was used to plot the similarity measures on a 2D planar space [62], with emotional concepts represented as nodes and their distance in space reflecting (inverse) similarity. The Kamada–Kawai algorithm is a force-directed graph-drawing algorithm that considers edges as springs and distances as spring rest distances. The algorithm finds a stable planar configuration, which we plotted in Fig 3A (displaying the topographic map for the pre-pandemic sample) and 3B (displaying the map for the pandemic sample). The nodes are marked according to normative measures of arousal and valence [50], their size represents the Arousal (big for high arousal, small for low arousal), and the color represents the Valence (blue for pleasant and red for unpleasant).

In keeping with the results of Barca et al. (2022) [17], valence and arousal were confirmed to be critical dimensions of the conceptual organization of emotions, both in the pre-pandemic period (Fig 3A) and in the pandemic period (Fig 3B). In both cases, emotional concepts were organized quite uniformly around the two axes of valence and arousal, without generating specific clusters, and into four quadrants formed by the two almost orthogonal axes (with a 94° angle in the pre-pandemic and a 96° angle in the pandemic dataset). Thus, the overall organization of the emotional concepts during the pandemic period was coherent with that of the pre-pandemic period, which may indicate a general stability of the core features of the conceptual knowledge of the affective domain. This is notable, considering the differences in the sample of participants and task methodologies between the two studies (e.g., laboratory vs. online task, mouse-tracking vs. mobile software).

Yet, some differences between the topographical maps of the two periods can be noted. Fig 3C shows that some emotional concepts were located differently between the two maps. Most of these concepts have *low arousal* and *positive valence* (i.e., ’astonished’, ’nice’, ’carefree’, ’respectful’, ’relaxed’, and ’reserved’), except for ’anxious’, which is a concept with high arousal and negative valence. Further, we found the correlation of concepts with the arousal axis to be greatly reduced in the pandemic dataset, suggesting a sloppier alignment of the concepts along the arousal dimension (Fig 3D). We also found that in the pandemic dataset, participants misclassified more concepts along both valence and arousal dimensions (Fig 3E), and there is lower entropy in the arousal dimension (Fig 3F), which indicates that the concepts span a smaller portion of space in the map.

Additional analyses were conducted to examine whether some characteristics of the population, such as gender or demographic characteristics, affect the conceptual representation of the affective domain during the pandemic period.

#### 3.4.1 Gender-related differences in the maps of emotional concepts

**Fig 4:**
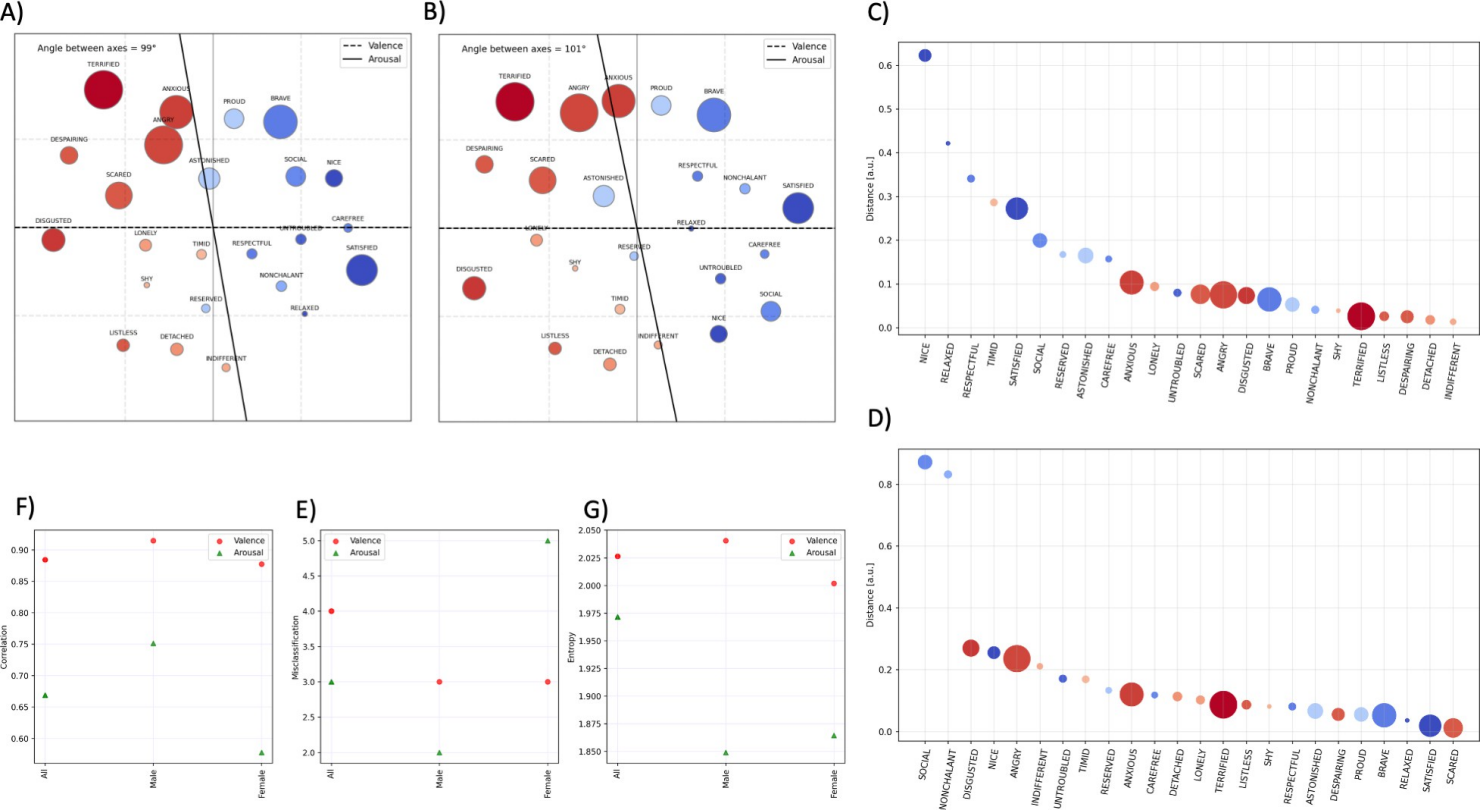
Two-dimensional map of emotional concepts of male (panel A) and female (panel B) Italian participants. The following indexes were calculated for the maps of different participant groups: *All participants*, *Males*, and *Females*. Panel C-D: Distance between the location of each emotional concept in the map of the whole participant group and the map of the (C) male and (D) female group. Panel E: Pearson correlations between the scores of valence or arousal and the projections on the two respective axes. Panel F: Misclassification of emotional concepts for the different participant groups. Panel G: Entropy of the concepts across the valence and arousal dimensions.

We investigated gender-related differences in the maps of emotional concepts formed by male (Fig 4A) and female (Fig 4B) participants. Since the sample of participants included more females (54) than males (35), we implemented a filtering process based on participants’ average Response Time (RT). We set a threshold of RT = 1.5 seconds, thus excluding participants with lower average Response Times. Our final dataset comprised 47 female and 26 male participants. To avoid biases due to the different sample sizes in the two groups, we reweighted the distances in Fig 3C and 3D based on the respective numerosity of the sample

$$\left(d_{male-all\;reweighted}=d_{male-all}*n_{all}/n_{male},d_{female-all\;reweighted}=d_{female-all}*n_{all}/n_{female}\right).$$

Compared to the map of the whole sample of participants (shown in Fig 3B), the map of the *male subgroup* reveals five main things. First, the angle between the two axes is substantially similar (i.e., 96° in the full sample, 99° in males). Second, the correlation between the scores and the projection on the axis increases for male participants, indicating a more consistent organization along both the valence and the arousal dimensions (Fig 4E). Third, male participants showed a reduced number of misclassification, across both valence and arousal dimensions (Fig 4F). Fourth, the map of the male group is less entropic (i.e., nodes span a smaller portion of the space) along the arousal dimension compared to the whole participant group (Fig 4G). Finally, the most significant differences between the placements of emotional concepts in the maps of male participants compared to the whole sample were for concepts with reduced arousal (e.g., ‘nice’, ‘relaxed’, ‘respectful’, ‘timid’), see Fig 4C.

Compared to the map of the whole sample of participants (shown in Fig 3B), the map of the *female subgroup* reveals five main things. First, the angle between the two axes slightly increases for female participants (101°) compared to the whole participant group (96°). Second, the correlation between the scores of arousal and the projection on the arousal axis decreases for female participants (Fig 4E), indicating a reduced coherence in the organization of the concepts along such dimension. Third, female participants misclassified more concepts along the arousal dimension (Fig 4F). Finally, the entropy of the map of female participants was reduced, particularly along the arousal dimension (Fig 4G), indicating that the concepts are less scattered in arousal space. Finally, the concepts that show the greatest differences between the maps of female participants and the whole sample are ‘social’ and ‘nonchalant’, which inverted their positions in the two maps (i.e., in the whole group, ’social’ is placed in the upper quadrant and ‘nonchalant’ in the lower quadrant, the opposite in the female subgroup), see Fig 4D.

In sum, the metrics of the map of the female subgroup indicate differences in the organization of emotional concepts at the level of the arousal dimension (compared to the whole group). Such gender-related differences in the affective knowledge representation–that parallels differences in the affectivity and interoceptive awareness self-report measures discussed above ‐ were not present in the pre-pandemic period, therefore we assume that they might be due to the pandemic-related circumstances.

#### 3.4.2 Demographic differences in the maps of emotional concepts

**Fig 5:**
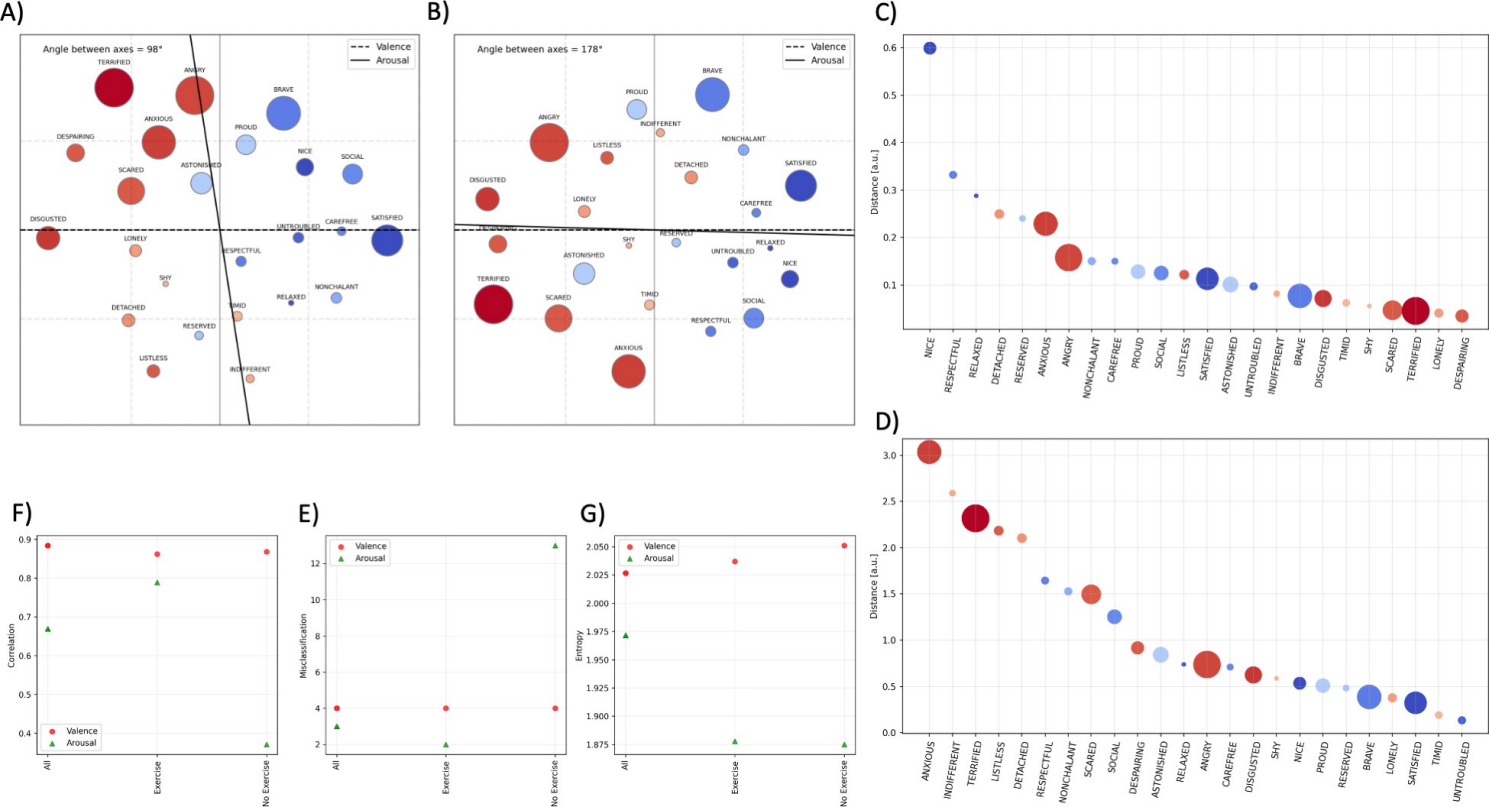
Two-dimensional map of emotional concepts of participants who performed physical activity (panel A) and those who did not (panel B). The following indexes were calculated for the maps of different participant groups: *All participants*, *Physical Activity*, and *No Physical Activity*. Panel C-D: Distance between the location of each emotional concept in the map of the whole participant group and the map of the (C) Physical Activity Yes and (D) Physical Activity No. Panel E: Pearson correlations between the scores of valence or arousal and the projections on the two respective axes. Panel F: Misclassification of emotional concepts for the different participant groups. Panel G: Entropy of the concepts across the valence and arousal dimensions.

Finally, we explored possible differences based on the demographics of the participants. Among all the demographic indexes that we considered in our questionnaires, we only found relevant differences between participants who performed physical activity (Fig 5A) versus those who did not perform physical activity (Fig 5B). Similar to the analysis of gender differences, the data of the two subgroups (19 Physical Activity Yes, 54 Physical Activity No) were normalized, to make them comparable with the whole sample.

The map (Fig 5A) and metrics of the participants who practiced physical activity (Fig 5E–5G) were overall similar to the data for the whole group (Fig 3B), with only some concepts being positioned differently, typically emotions with positive valence and low arousal (i.e., ‘nice’, ‘respectful’, ‘relaxed’, ‘detached’, ‘reserved’).

Rather, a comparison of data from the whole group (Fig 3B) and the subgroup of participants who did not practice physical activity (Fig 5B) showed more significant differences. This is the only subgroup in which the orthogonality between the valence and arousal axes is lost; this is evident by considering that the angle between the two dimensions in Fig 5B is 178°. This is due to a collapse of the arousal axis, with an undifferentiated distribution of concepts between the upper and lower quadrants of high and low arousal. As a further consequence of this collapse, the metrics of the map of participants who did not practice physical activity (Fig 5E–5G) are very different from the whole sample: the correlation of concepts with the arousal axis is considerably reduced (Fig 5E); the number of misclassifications of arousal concepts is much larger (Fig 5F) and concepts are more grouped within the arousal dimension, as indexed by lower entropy (Fig 5G). Finally, in the map of participants who did not practice physical activity, many emotional concepts (16 out of 24) are misplaced compared to the whole sample (Fig 5D; please consider that for better readability, the scale of this panel is much wider than the other equivalent panels). The shift occurs on concepts with negative valence and with low arousal (except for ’anxious’, ’terrified’, ‘scared’, and ‘angry’), but also on concepts with positive valence and low arousal (i.e., ‘respectful’, ‘nonchalant’, ‘social’, ‘astonished’, ‘relaxed’).

It is worth considering that most of the participants in the subgroup of people who did not perform physical activity were female (79%), aged between 30 and 50 years (80%) (more specifically: 20% females are aged 18 years, 13% aged 29 years, 47% aged 39 years, 7% aged 49 years and 13% aged 59 years). Considering the family composition, the majority of the subgroup (11 out of 19) lived alone with a child. Most had a garden (15 out of 19) and their working condition was quite varied (6 had permanent employment, 1 had a fixed-term position, 4 were freelancers, 1 was a university student, 2 were unemployed, 4 with ’other’, and 1 missing case). As for their geographical location, they were mainly residents in cities in central Italy (68%). While any combination of these factors might be relevant to our results, the most prominent characteristic of this subgroup appears to be the fact that it is predominantly composed of middle-aged females (we will return to this point in the following section).

## 4. Discussion

This study asks whether the detrimental effects of the COVID-19 pandemic on the emotional distress of the Italian population (the hardest-hit European country) extend one year after the outbreak–and it reports that this is indeed the case. Specifically, this study reveals three main findings.

First, the sample population, which was tested 14 months after the most critical phase of the health emergency, experienced negative affectivity (being ‘nervous’, ‘irritable,’ and ‘scared’) to a greater extent than a sample tested in a pre-pandemic period [17]. These results align with previous studies conducted in Italy [4] and other countries at the beginning of the pandemic [7, 8], therefore we believe they might be generalized to other populations. The negative affectivity and the variability across subjects increased on average. The enhancement of negative affectivity was present in both males and females, but in the case of females, we cannot accept or reject the null value with confidence as there is not enough precision in the estimate. Previous studies reported that, in typical conditions, females score higher than males on the negative affectivity scale [51, 63]. Since we failed to report gender differences before the pandemic [17], the greater observed variability in the female pandemic sample might point to a heightened vulnerability of female participants to the stressors associated with the pandemic [64], which lasts beyond the state of the emergency. The differences in positive affectivity, instead, were reduced across the pre-pandemic and pandemic periods and the estimate was uncertain. On a general note, this confirms that the positive and negative affectivity measures of the PANAS are highly distinctive dimensions [65, 66]. Since the outbreak of the pandemic low levels of positive affectivity have been reported [7]. The current result reassuringly may indicate a re-establishment of positive emotionality and a phase of well-being recovery in the population (see also Bourmistrova et al., 2022 for similar results on measures of anxiety and depression) [67]. It is possible that the easing of the containment measures and the development of coping strategies might have contributed to regaining some of the core features of positive affectivity, such as having interest and the ability to experience pleasant emotionality. However, it is worth noticing that parameters estimation was uncertain and these observations on positive affectivity need further testing.

Second, the self-report measure of *awareness of inner bodily sensations* showed some differences between the pre-pandemic and the pandemic periods. In particular, the pattern of the MAIA subscales’ inter-correlation and the strength of the correlations increased substantially during the pandemic period. We were particularly interested in the Noticing subscale because its’ high values have been associated with maladaptive aspects of interoceptive sensibility [68] and increased anxiety and depression during mild lockdown [35]. Interestingly, we reported a *positive relationship* between the awareness of body sensations and adaptive aspects of interoceptive sensibility, such as mindful attention to interoceptive cues and emotion regulation. Therefore, unlike what was reported in the early stages of the pandemic [35], about a year later we have no evidence of heightened maladaptive aspects of interoception. As noted earlier, the observed phenomenon could be explained by the emergence of specific coping mechanisms.

Another important finding that emerged during the pandemic, and which we had not observed before [17], is the presence of gender-related differences, with a greater tendency of females to consciously sense inner bodily signals. Gender-related differences in interoceptive processing have previously been questioned because of mixed results, partly due to the different facets of interoception. For example, when considering *interoceptive accuracy* (i.e., the objective accurate detection of bodily sensations) [69] some studies reported that males outperform females in accurately perceiving their heartbeat [70, 71], which have been recently questioned by a newly developed measure of cardioception not affected by physiological or strategic confound [72]. Addressing gender-related differences in the perception of interoceptive signals other than the cardiac ones (e.g., gastric and respiratory signals) is even more complicated by methodological factors that contribute to the emergence of conflicting results [71]. As for the *awareness* of bodily sensations (typically measured with the MAIA), some reports that females had higher scores than males on several different subscales [70]. Notably, we did not find reliable evidence of gender-related differences in interoceptive awareness before the pandemic [17] but during the pandemic we found credible results. About a year after the outbreak, females have high scores, particularly on two features of interoceptive awareness: the ability to attend to inner bodily sensations *(Attention Regulation*) and the awareness of physical sensations associated with emotions *(Emotional Awareness*). Those subscales have been found to positively correlate with ‘*emotional susceptibility*, or the tendency to experience feelings of discomfort and vulnerability when facing emotionally-laden stimuli [52]. In addition, individuals with high emotional awareness showed stronger emotional reactivity [73] and higher scores in measures of trait anxiety [19]. Heightened interoceptive awareness (at cardioception) is associated with enhanced physiological reactivity to emotional stimuli [74] and anxiety disorders [75]. In light of this evidence, we speculate that the overall increase in interoceptive awareness of females during the pandemic might be related to a greater emotional susceptibility, which is related to greater variability in self-reported affectivity (consistent, for example, with the results of negative affectivity of the PANAS) and impacts on the autonomic activity [76]. Albeit speculative, this explanation is consistent with theoretical models of (mis)perception of bodily signals suggesting that–in some conditions–greater attention to inner bodily sensations might be problematic [29, 30]. Likewise, heightened emotional awareness has been associated with emotion dysregulation (i.e., experiencing negative stimuli with greater intensity and greater physiological activation) in atypical populations, as patients with somatic syndrome disorders compared to controls [76].

Third, the effect of the pandemic-related conditions manifests in changes in *affective knowledge representation*. To assess this, we used the similarity judgment data of this study to derive a 2-dimensional map of emotional concepts and we compared it with the pre-pandemic map reported in Barca et al., (2022) [17]. The fundamental principles of organization of concepts around the two orthogonal axes of valence and arousal were confirmed, suggesting general stability of the core features of the conceptual knowledge of the affective domain (see also Kring et al., 2003; Russell, 1980) [42, 77]. This is notable, considering the differences in the study sample and task methodologies (i.e., laboratory vs. online task, mouse-tracking vs. mobile software). Among the two axes of the maps of emotions, the affective valence dimension was confirmed to be more stable across pre-pandemic and pandemic periods, whereas we found a greater malleability of the arousal dimension, as highlighted by a reduced correlation between the emotional concepts and the arousal axis in the pandemic map.

Additional fine-grained analyses revealed interesting gender differences. Specifically, the map of female participants was characterized by a reduced coherence in the organization of the concepts along the arousal dimension and by more errors in the classification of concepts based on arousal. These results parallel the gender-related differences that we found in interoceptive sensibility and suggest a sloppier representation of the arousal- and physiological-related features of emotional concepts, particularly those referring to social and well-being factors.

Furthermore, we analyzed how demographic characteristics influence the mental maps of emotional concepts. We found that the map of the subgroup of participants who do not practice *physical activity* showed a collapse of the arousal dimension with a high number of errors in the placement of concepts with low arousal, regardless of positive or negative affective valence. The mislocated concepts pertain to the social sphere–possibly suggesting the need for social proximity when social distancing was no longer mandatory but still recommended ‐ and an anxious/scared emotionality. This result was not reported before but it is not entirely surprising, given that several studies converge in pointing out the beneficial effects of performing physical activity. Considering mood disorders, performing physical activity concurs in the reduction of symptoms of depression and anxiety, acting on both psychological mechanisms (e.g., by diverting from unpleasant stimuli, or by increasing the sense of self-efficacy) and physiological mechanisms (e.g., through the release of monoamines and endorphins, which seem to alleviate these negative emotionalities; see Paluska & Schwenk (2000) for a review) [78]. The positive effects of performing physical activity go beyond psychiatric disorders, extending also to a variety of chronic conditions, such as neurological, metabolic, cardiovascular, and pulmonary diseases [79]. Aerobic activity (i.e., running) has been tested as an ‘interoceptive exposure technique’ within a cognitive-behavioral intervention to reduce anxiety sensitivity [80]. Despite some initially promising results, this exposure technique does not seem to have been further evaluated. Wallman-Jones et al., (2021) [81] address in detail the relationship between physical activity and interoceptive processing, and suggest that the two are linked by a feedback loop mechanism. Bridging the literature on exercise regulation and interoception, they suggest that if on the one hand, more efficient processing of bodily signals (such as fatigue and effort levels) might be beneficial in practicing physical activity, physical activity could potentially circularly influence interoceptive processes. Since the majority of our population sample practiced some physical activity, this might have been a *protective factor* against the stressful pandemic-related conditions. Lacking this protective factor, the subgroup of those who do not practice physical activity might have been more exposed to the pandemic stressors even beyond the outbreak. However, this speculative hypothesis remains to be fully tested in future studies.

Additional susceptibilities might be related to *age-dependent gender differences*. Most of the participants who did not perform physical activity were females, who overall (whether they practice physical activity or not) exhibited greater vulnerability than males in the measures of affectivity and awareness of body signals that we have considered. Furthermore, most of them were middle-aged women and might be experiencing endocrinological changes related to the menopausal transition.

Bearing in mind that we have no confirmation that these women are experiencing the menopause transition, we develop this hypothesis with all the speculative caveats, deferring to future targeted empirical validations.

The menopause transition might have a large impact on a woman’s life with enhanced susceptibility to affectivity and developing health issues [82]. Perimenopause and menopause by themselves do not necessarily increase the risk of developing mood disorders [83], except in women with a history of anxiety and depression [84]. However, several modifications take place leading to an increase in vasomotor symptoms (which are uncomfortable and might have a secondary effect on well-being) and fluctuation in estrogen regulation [84] which is strongly related to affectivity (e.g., some of the brain regions centrally involved in mood regulation (such as the amygdala, hippocampus, and hypothalamus) are sensitive to the fluctuating levels of estrogen). Prentice & Murphy (2022) [71] address this issue specifically, and suggest that the dramatic physiological changes that women experience during their life (e.g., menstruation, pregnancy, lactation, and menopause) might account for the increased incidence of psychiatric disorders in women, and which manifest in enhanced attention to bodily signals coupled with poor accuracy in perceiving them. The discrepancy between different facets of interoception (as accuracy and sensitivity) is a good predictor of elevated levels of anxiety and emotional deficits in clinical populations [85].

Thus, hormonal fluctuations occurring in the menopausal transition might hamper interoceptive processes making uncertain the reference to inner bodily information and, consequently, the reference to physiological arousal as a critical dimension in affective knowledge representation. This is a speculative hypothesis that requires future studies to be validated.

### 4.1 Limitations of the study

In this study, we used a dataset collected before the pandemic [17] as a benchmark for the performance of a population sample obtained one year after the outbreak. The recruitment process, experimental setting, and composition of the two population samples were (necessarily) different due to contextual conditions. From a methodological point of view, for example, the pre-pandemic sample executed the experiment in a laboratory setting, via computer, and was enrolled through in-person advertisements. This group comprises 30 participants, carefully balanced by gender and age. Differently, the pandemic sample executed the experiment online via mobile phone and was (necessarily) recruited online. It comprises 89 participants, the majority of female gender, and with a wider age range. Therefore the pre-pandemic sample is not a carefully matched control group, and it remains to be established to what extent these differences in recruitment, experimental setting and composition of the two groups affect the generalizability of the results.

Another potential issue is the gender bias of the pandemic population sample, which was mainly composed of female participants. Although we designed it as a cross-sectional study, the online recruitment of participants of the pandemic sample may have induced a bias in participant engagement. Typically, online enrolment methods (via social media or website) are more efficient than offline methods (e.g., flyers, posters) in recruiting a higher number of participants, but might be more suitable for cohort studies [86] as some bias might emerge limiting the generalizability of the study [87]. Some mitigation strategies allow for improving the diversity of the sample population, considering for example race or ethnicity [88]. Nevertheless, it is worth noticing that online recruitment often exhibits a disproportion in participants’ gender (see for example, Cali’ et al., 2015) [52], which resulted be particularly common during the pandemic (for example Bacaro et al., 2020; Eichenberg et al., 2021; Fiorillo et al., 2020; Modzelewska et al., 2021; Montefinese et al., 2021; Suzuky et al. 2021) [6, 5, 13, 35, 50], and might represent a greater willingness of the female gender to participate in online studies during the pandemic. For these reasons, we are careful not to overstate the strength of our findings and we emphasize the need for further research to replicate and extend our results.

## 5. Conclusions

Our study aims to contribute to a better understanding of how individuals represent and experience emotions and interoception, and provides insights on how the COVID-19 pandemic may have affected people’s conceptual representation of emotions and interoceptive experiences. It is among the few that investigate the prolonged impact of a major global crisis on the affectivity of the population. By using a combination of methods (encompassing pre-pandemic data as a benchmark, self-report questionnaires, and experimental tasks), this study provides a comprehensive account of how conceptual representations of emotions–and associated interoceptive processes–changed before and after the pandemic. It makes several novel contributions to the existing body of knowledge on conceptual representations and interoception, focusing on the long-term effects of the COVID-19 pandemic.

The most important result of the study is showing that the effects of the pandemic persist–albeit possibly mitigated–about a year after the outbreak and manifest especially in the female gender, which has been associated with heightened psychological distress since the beginning of the pandemic [89]. Males show an increase in negative affectivity, which is not accompanied by differences in interoceptive sensitivity or in affective knowledge representation. Taken together, the measures of positive and negative affectivity, interoceptive awareness, and a new measure we recently developed to explore affective knowledge representation [17] suggest a greater emotional susceptibility of females. Therefore, importantly, the study emphasizes the role of gender and individual differences in shaping conceptual representations, highlighting the interplay between personal factors and broader social contexts in shaping affectivity in response to a global crisis.

Furthermore, our findings contribute to advancing theoretical frameworks in psychology, cognitive science, and social neuroscience by providing insights into the dynamic nature and malleability of conceptual representations and the factors that influence their formation and modification. Indeed, our results align well with constructivist views (Barrett, 2017; 2018) in showing that individual experiences–beliefs, values, and sociocultural factors–and shared cultural contexts can influence conceptual representations of emotions and associated interoceptive processing.

Finally, our findings have implications for understanding the impact of global crises on mental health and (potentially) for developing targeted interventions that promote resilience in the face of challenges. Our findings advise providing programs of psychological and emotional assistance to the population throughout the pandemic period and beyond, and that age-dependent gender differences should be accounted for to define tailored interventions for individuals experiencing difficulties with emotional regulation or interoceptive awareness. For instance, interventions could focus on enhancing emotional differentiation, improving interoceptive accuracy, or fostering adaptive coping strategies. Interestingly, physical activity emerged as a potential protective factor in relieving pandemic-related stressors, so–whenever possible–it should be considered among the activities allowed and scheduled during particularly stressful periods for the population. These are just potential applications of our findings, and further research is needed to fully explore their clinical implications. We hope that our study can serve as a foundation for future research that will ultimately benefit individuals struggling with emotional and interoceptive challenges.

## Supporting information

S1 Appendix (DOCX)
