# Supplementary material for: Emotional distress and affective knowledge representation one year after the COVID-19 outbreak
Source: PLoS One. 2025 Jan 17;20(1):e0311009. doi: 10.1371/journal.pone.0311009 (PMC11741616; doi:10.1371/journal.pone.0311009)
Supplement: S1 Appendix — (DOCX) [file pone.0311009.s001.docx]

**Appendix.**

1. **Results of Bayesian parameter estimation on PANAS scores**

*1.1. Positive affectivity in the pandemic and pre-pandemic samples.*

Bayesian parameter estimation (Kruschke, 2011; Meredith and Kruschke, 2021) indicates that the difference between the mean of the derived parameter for the positive scores (muDiff) is on average 0.106, and the majority of the credible values (71%) is greater than zero (that is, the probability that the true value is greater than zero is large). The mean difference in standard deviations of the derived parameters (sigmaDiff) is 0.08 and the probability that the true value is greater than zero is shown at 74%. Both the mean and standard deviation of the scores of the pandemic group are credibly larger than the values of the pre-pandemic group, meaning that the positive affectivity scores increased on average, but also the variability. However, inspection of the posterior distribution reveals a great uncertainty in the estimate of the differences in means and standard deviations, as in both cases a difference of zero falls within the 95% HDI (a reduced precision in the estimate that might be due to the limited sample size). The distribution of credible effect sizes has a mode of 0.29 and about 14% of the credible values fall with a ROPE defined around a small effect size.

*1.2. Negative affectivity in the pandemic and pre-pandemic samples.*

The average difference in the mean of the derived parameters for the negative scores is muDiff = 0.66. Almost the entire distribution of the credible values is greater than zero (99%) and, considering the posterior distribution, 95% HDI of the differences in means falls well above zero. Therefore is it possible to conclude that the means of the two groups are credibly different (specifically that the scores of the ​ pandemic sample are higher than those of the pre-pandemic sample), with some degree of certainty. The average difference in standard deviations of the derived parameters (sigmaDiff) is 0.28, the 96% of the credible values are greater than zero. The posterior distribution confirms a greater standard deviation of the derived parameters of the pandemic sample (a difference of zero falls above the 95% HDI). The distribution of credible effect sizes has a mode of 1.6 and about 0.29 of the credible values fall within a ROPE defined around a small effect size. To summarize, Bayesian estimation suggests a credible difference in self-reported affectivity ​​of the pandemic and pre-pandemic, which is more marked and reliable on the negative affectivity subscale (as shown by the effect size). The analysis also indicates greater variability in the scores of the pandemic sample.

*1.3 Positive affectivity of male participants of the pandemic and pre-pandemic samples.*

Taking into account the scores of positive affectivity of the male subgroup, the average difference in the means of the derived parameters is rather small (muDiff = 0.02) with only 53% of the credible values greater than zero. The 95% HDI of the differences in means is centered around zero, leading to the conclusion that the means of the two groups are not credibly different. Similarly, the average difference in standard deviations sigmaDiff is 0.02 and 55% of the credible values are greater than zero. The posterior distribution shows great uncertainty in the estimate of the difference in standard deviations, such that a difference of zero falls within the 95% HDI. The distribution of credible effect sizes has a mode of 0.04 and about 17% of the credible values fall within a ROPE defined around a small effect size.

*1.4 Positive affectivity of female participants of the pandemic and pre-pandemic samples.*

In the *female subgroup*, the average difference in the mean is muDiff = .21, and 85% of the credible values are greater than zero, but the posterior distribution shows great uncertainty in the estimate (95% HDI of the differences in means includes the zero), so is not possible to conclude that the means of the two groups are credibly different. The average difference in standard deviations (sigmaDiff) is 0.09 and 77% of the credible values are greater than zero. However, the posterior distribution shows great uncertainty in the estimate of the difference in standard deviations, such that a difference of zero falls within the 95% HDI. The distribution of credible effect sizes has a mode of 0.48 and about 10% of the credible values fall within a ROPE defined around a small effect size.

*1.4 Negative affectivity of male participants of the pandemic and pre-pandemic samples.*

As for the negative affectivity of the male subgroup, the average difference in the mean of the derived parameters is muDiff = 0.81, the vast majority of the credible values (99.9%), and 95% HDI of the differences in means fall above zero. The average difference in standard deviations of the derived parameters (sigmaDiff) is 0.39, the majority of the credible values are greater than zero (99.7%), and 95% HDI of the differences falls above zero. Thus, both the means and standard deviation of the two groups are credibly different. The distribution of credible effect sizes has a mode of 2.03 and a small percentage of credible values (0.1%) fall within a ROPE defined around a small effect size, suggesting an effect size greater than -0.1 to 0.1.

*1.5 Negative affectivity of female participants of the pandemic and pre-pandemic samples.*

In the case of the *female subgroup*, the difference in the mean (muDiff) of the derived parameters is on average 0.52. The 95% HDI of the differences in means falls well above zero, and 99% of the credible values are greater than zero, therefore we conclude that the means of the two groups are credibly different. The difference in standard deviations of the derived parameters (sigmaDiff) is 0.13 and 82% of the credible values are greater than zero. However, the posterior distribution shows great uncertainty in the estimate of the difference in standard deviations, as a difference of zero falls within the 95% HDI. The distribution of credible effect sizes has a mode of 1.17 and about 1.3% of the credible values fall within a ROPE defined around a small effect size.

1. **Results of Bayesian parameter estimation on MAIA scores**

*2.1 Interoceptive awareness in the pandemic and pre-pandemic samples.*

The average mean difference of the derived parameter for the MAIA score of the pandemic and pre-pandemic sample is muDiff = 0.09. The majority of the credible values (83%) is greater than zero, but the posterior distribution indicates uncertainty in the estimates (zero is included within the 95% HDI). The difference in the estimates of the standard deviations’ parameters (sigmaDiff = -0.06, with a 20% probability that the true value is greater than zero) is not credible, and the sample size computation (mode = 0.25, and 20% of credible values within the ROPE). Overall, parameter estimation results are very uncertain and we cannot draw firm conclusions.

*2.2 Interoceptive awareness of females and males of the pandemic sample*

Considering the gender of the pandemic sample, the average mean difference of the derived parameter for females and males is muDiff = 0.28, and the vast majority of credible values is greater than zero (99.8%). The posterior distribution supports the credibility of the difference (the 95% HDI falls above zero). The mean difference in standard deviation is small (sigmaDiff = 0.01). Only 54% of the credible values ​​are greater than zero, and the zero falls within the 95% HDI suggesting great uncertainty in the estimate. The distribution of credible effect sizes has a mode of 0.75 and about 0.54% of the credible values fall within a ROPE defined around a small effect size. The results suggest that, overall, during the pandemic female participants have higher MAIA scores than males, however, the estimate of the variability has a high uncertainty.

*2.3 Interoceptive awareness of females and males of the pre-pandemic sample*

In the pre-pandemic sample, the average mean difference of the derived parameter for females and males is muDiff = -0.15, and only 10% of credible values are greater than zero (the vast majority is smaller than zero, but the posterior distribution questions the credibility of the difference (the 95% HDI include the zero). The mean difference in standard deviation is small (sigmaDiff = 0.01). Only 56% of the credible values ​​are greater than zero, and the 95% HDI is centered around zero indicating great uncertainty in the estimate. The distribution of credible effect sizes has a mode of -0.33 and about 13% of the credible values fall within a ROPE defined around a small effect size. In the posterior distribution, the zero falls within the 95% HDI, suggesting uncertainty in the estimate. Thus, the results indicate a high uncertainty in the parameter estimation of the MAIA score of the pre-pandemic sample.

*2.4 Interoceptive awareness of males of the pandemic and pre-pandemic samples*

Bayesian parameter estimation of male scores of the pandemic and pre-pandemic samples did not yield credible results. The average mean difference of the derived parameter is muDiff = -0.15, and only 8% of credible values are greater than zero (the vast majority is smaller than zero), but the posterior distribution questions the credibility of the difference (the 95% HDI includes zero). The mean difference in standard deviation is small (sigmaDiff = -0.08), 14% of the credible values ​​are greater than zero, and the 95% HDI includes zero (and is not credible). The distribution of credible effect sizes has a mode of -0.38 and about 12% of the credible values fall within a ROPE defined around a small effect size. The value of zero falls within the 95% HDI of the posterior distribution, suggesting uncertainty in the estimate.

*2.5 Interoceptive awareness of females of the pandemic and pre-pandemic samples*

As for the females’ scores, the average mean difference of the derived parameter of the pandemic and pre-pandemic period is muDiff = 0.28, and the vast majority of credible values (99.5%) is greater than zero, with credibility from the posterior distribution. The mean difference in standard deviation is small (sigmaDiff = -0.09), 14% of the credible values ​​are greater than zero, and the 95% HDI includes zero suggesting uncertainty in the estimate. The distribution of credible effect sizes has a mode of 0.69 and about 12% of the credible values fall within a ROPE defined around a small effect size. The value of zero of the posterior distribution falls outside the 95% HDI, providing credibility to the estimate. Thus, parameter estimation indicates a credible difference in females’ MAIA scores, which are higher during the pandemic.
